# How Adolescents and Parents See Their Moral Responsibilities With Regard to Adolescents Using Alcohol—A Deductive Secondary Analysis

**DOI:** 10.1111/josh.13248

**Published:** 2022-10-17

**Authors:** Mari A. Mynttinen, Kaisa E. Mishina, Mari K. Kangasniemi

**Affiliations:** ^1^ Department of Nursing Science, Faculty of Medicine, University of Turku, 20014 Turku, Finland; Nursing Degree Program Karelia University of Applied Sciences 80200 Joensuu Finland; ^2^ Departments of Nursing Science and Child Psychiatry, Faculty of Medicine, University of Turku, 20014 Turku, Finland INVEST Research Flagship Center Turku Finland; ^3^ Department of Nursing Science, Faculty of Medicine University of Turku 20014 Turku Finland

**Keywords:** adolescents, alcohol, deductive secondary analysis, responsibility, parents

## Abstract

**BACKGROUND:**

This study described how adolescents and the parents saw their moral responsibilities with regard to adolescents using alcohol.

**METHODS:**

This was a deductive secondary analysis, based on Hart's taxonomy of moral responsibility. The primary studies were based on 19 group interviews with 87 adolescents aged 14‐16 and 17 interviews with 20 parents. Voluntary participants were recruited by purposive sampling from two public schools in Finland.

**RESULTS:**

Role responsibilities comprised of adolescents taking care of themselves and parents providing authority figures and helping adolescents to make rational decisions about alcohol. Capacity responsibilities referred to adolescents' abilities to make independent decisions on using alcohol and their developing abilities to control their actions. Parents required abilities to get involved in and show an interest in their children's everyday lives. Causal responsibilities focused on ensuring that adolescents did not cause harm when they used alcohol, and parents had to acknowledge and react to the consequences. Liability responsibilities were about the law on alcohol use and responsibilities for any legal consequences. The role schools could play was important.

**CONCLUSIONS:**

Adolescents and parents had wide‐ranging responsibilities related to the adolescents' using alcohol and school nurses could play an important role in healthy decisions.

Research on adolescent health promotion has increasingly paid more attention to the personal responsibility that adolescents take for their health choices. Responsibility refers to ethical or role‐based expectations for actions or consequences, based on which ethical activities can be assessed.^1^, ^2^, ^3^ The perspectives that adolescents have about their responsibilities for behavior that harms their health plays a key role in health promotion. Adolescents' health choices refer to the conscious or unconscious decisions they make in their everyday life, when they make their own decisions or are influenced by their parents, peers, or family. These choices are affected by their knowledge, skills, and the wider social environment.^4^, ^5^, ^6^ Relatively little research has been published on the perspectives of adolescents and their parents on various subjects, even though they are an essential part of health promotion. Preventive school health care services need more information, so that they can support discussions by adolescents and their parents about their responsibilities,^7^ to promote health literacy among adolescents^8^ and to encourage adolescents to take responsibility.

Adolescence takes place from 10 to 19 years of age^5^ and this study focused on adolescents aged 14‐16 years. An important part of growing up is that individuals need to take responsibility for their health, well‐being, and related choices.^9^, ^10^ Thinking and decision‐making skills are still developing during adolescence,^11^ which means that they may take risks and act impulsively.^12^ Health choices about nutrition, rest, physical activity, and using intoxicants, including alcohol,^5^ provide important foundations for their health and well‐being.^4^ Adolescents identify with their peers and they have a significant impact on the choices that they make.^13^ Their parents and home lives also continue to play a major role for adolescents.^14^, ^15^ Parents are responsible for safeguarding and promoting their child's well‐being,^16^ and they focus on education,^17^ decision‐making and caring for their child.^18^ Parents' socioeconomic status, attitudes, and educational background have been shown to affect the choices that adolescents make about using alcohol.^19^

Alcohol is still the most commonly substance used by adolescents,^5^, ^14^, ^20^ often before the age of 15.^5^ Global figures suggest that 155 million adolescents aged 15‐19 (27%) use alcohol^20^ and one European study found that 80% of adolescents aged 15 and 16 had tried alcohol.^21^ Alcohol may have a lasting impact on an adolescent's health and well‐being, as it has short‐term and long‐term consequences.^5^, ^6^, ^22^ This raises concerns about adolescents' health and well‐being.

Questions about moral responsibilities focus on the correctness, morality, or immorality of an individual's actions or inaction.^23^ In this study, moral responsibility has been divided into role responsibility, capacity responsibility, causal responsibility, and liability responsibility, according to Hart's taxonomy.^24^ Role responsibility refers to the duties a person performs, based on their authority and status, to promote the well‐being and objectives of themselves or other people. Capacity responsibility refers to an individual's capacity to negotiate, make, and adhere to decisions and control their functioning. Causal responsibility refers to being responsible for one's own or another person's actions and their consequences and liability responsibility refers to a moral responsibility to follow the law. Hart's taxonomy has been used to examine the moral responsibilities of health care patients who make avoidable mistakes^1^ and investigate depression as an explanatory factor for immoral behavior.^25^ To the best of our knowledge, Hart's taxonomy has not been used in empirical nursing science research.

Discussions with adults about moral and legal perspectives may stop adolescents from starting to use alcohol.^7^ Adolescents have described their responsibilities as measures taken to promote their own health, or other people's health, including avoiding substances and alcohol.^15^, ^26^ Studies have reported that adolescents who stuck to their personal moral rules felt that intoxication was wrong^7^, ^27^ and their peers found it hard to influence them.^27^ Parents have said that they were responsible for providing guidance, monitoring and protection, setting rules, and providing information to ensure that adolescents avoided the harmful effects of using alcohol.^14^, ^28^ The parents' own attitudes and alcohol cultures affect how their adolescents experiment with alcohol. Alcohol use can be reduced by parents taking responsibility and getting involved in the lives and health of their adolescents.^29^, ^30^ The views of adolescents and parents about responsibilities form part of a topical research subject with ethical justifications.^15^, ^23^, ^31^

The aim of this study was to describe how adolescents and parents saw their moral responsibilities with regard to adolescents using alcohol and how these responsibilities were related to each other.

## DESIGN AND METHODS

**Figure 1:**
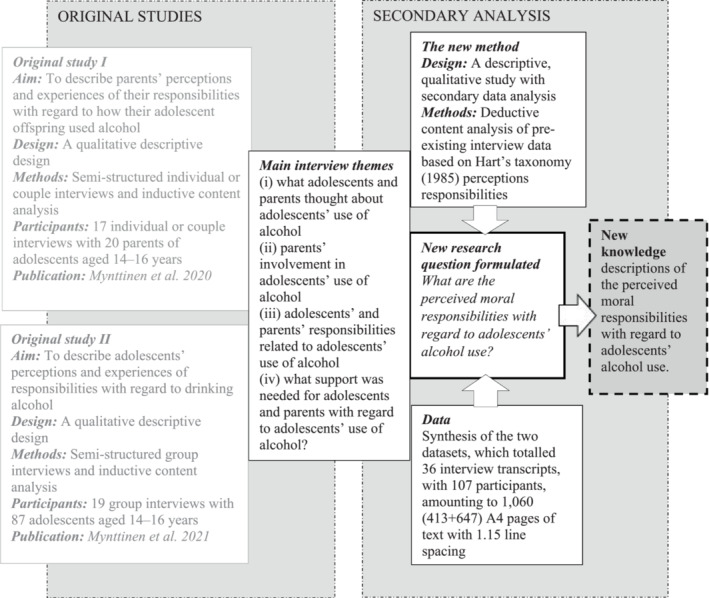
The Study Process

This was a deductive secondary analysis that explored the original datasets^28^, ^32^ of two studies conducted in Eastern Finland in 2017 (Figure 1). We used the consolidated criteria for qualitative research to ensure explicit and comprehensive reporting of the study and to improve its rigor, comprehensiveness, and credibility.^33^ The method enabled us to reanalyze large datasets that were originally collected during primary studies^34^, ^35^ and deepen and enrich them by generating new knowledge.^36^

### Recruitment and Participants

The participants were recruited from one urban and one rural public secondary school and purposive sampling was used to conduct the interviews. The researcher (M.A.M.) obtained permission from the school districts and approval from the Committee on Research Ethics of the University of Eastern Finland. Then, the head teachers were contacted for their permission to recruit participants and they emailed information letters to ninth‐grade students, aged 14‐16 years, and their parents. The researcher visited the schools and presented the study aims and process. The adolescents were invited to take part in group interviews during school days and were given written copies of these emailed letters to take home. These letters invited their parents to participate in individual interviews in a place of their choice, such as at home or in public libraries. Five of the parents were recruited using a snowballing technique, with the help of other participating parents.^37^ The interviews were carried out once informed consent had been received from the voluntary participants.

### Data Collection

We combined the cross‐sectional data from 36 interview transcripts. This covered 19 group interviews with 87 adolescents aged 14‐16 years old and interviews with 14 individual parents and three couples. The participants were not members of the same family. Each interview used the same semi‐structured interview guide^38^ and the four main themes were based on previous studies (Figure 1). The interviews were audio recorded and transcribed verbatim.

### Procedures for Coding Qualitative Data

We used deductive secondary analysis to describe the responsibilities in percentage terms,^39^ so that we could quantify the number of views on various aspects of responsibilities. We developed a broad conceptual framework by identifying four large main categories for the data‐driven deductive analysis in accordance with Hart's taxonomy.^24^ The role responsibilities category comprised any views that focused on the tasks that needed to be performed by the adolescents and parents. Capacity responsibilities focused on abilities or resources, such as time and willingness. Causal responsibilities related to views on the consequences of alcohol use. Liability responsibilities focused on the law.

The researcher (M.A.M.) entered each transcribed interview into NVivo 12 Plus coding software and reviewed each one. Although the views that were expressed covered diverse themes, most of them could be roughly classified under the four main Hart taxonomy categories. The content of the categories had not been predetermined. Then, the researcher allocated codes to the detailed content and classified them into subcategories based on their similarities and differences. These subcategories then formed the basis for the four final categories.^40^ Some of the codes could fall into more than one category and the full research team discussed how they could improve the accuracy of the categories and develop a final dictionary of codes. For example, parents were responsible for compensating for the damage cause by their adolescent child using alcohol, and this came under both causal responsibilities and liability responsibilities. However, each code was only categorized once. Code saturation was reached when the research team reached a consensus that the codes were adequately represented in the relevant categories.^41^ The subcategories and categories were partly redefined and renamed to reflect the final representation of the codes. The numbers of defined and named subcategories and categories were displayed, which helped the research team to quantify and compare the views that had been expressed. The percentage of the codes in each category were calculated in relation to all the views that addressed that particular question.

### Ethical Considerations

The school district and the two participating schools provided permission for their students to take part. The Committee on Research Ethics of the University of Eastern Finland (Statement UEF/12/2017) granted ethical approval for the study. The information letter described the aims, privacy, and confidentiality of the study, the voluntary nature of the participation and stated that students and parents could withdraw at any time. Participants gave their oral and written informed consent to participate.^42^ There were 80 students aged 15 and 16 who were able to provide consent, according to the ethical principles of research^43^ and Finnish law.^44^ The seven students aged 14 returned the consent forms signed by their parents. Written consent was necessary to allow us to analyze the anonymized interview data. We stressed that nothing that was discussed would be disclosed to third parties. The study followed the research ethics principles in the Declaration of Helsinki and responsible research practice.^43^

## RESULTS

### Characteristics of the Participants

A total of 87 adolescents and 20 parents participated in the interviews (Table 1) and their mean ages were 15 and 46 years, respectively. Most of the participants had 4‐5 family members and most of them lived in rural areas.

**Table 1:** Characteristics of the Participants

All participants (N = 107)	Adolescents (N = 87, 81%)	Parents (N = 20, 19%)
Female	50 (57)	13 (65)
Male	37 (43)	7 (35)
Age in years		
14	7 (8)	
15	71 (82)	
16	9 (10)	
30–39		5 (25)
40–49		8 (40)
50–59		6 (30)
60+		1 (5)
Number of family members		
2–3	13 (15)	1 (5)
4–5	52 (60)	11 (55)
6–7	11 (13)	3 (15)
8–9	5 (6)	0 0
10–12	5 (6)	0 0
School area		
Rural area	76 (87)	16 (80)
Urban area	11 (13)	4 (20)
Degree/education		
Academic		4 (20)
College		9 (45)
Comprehensive/high school/vocational		7 (35)

### The Senses of Responsibilities

**Figure 2:**
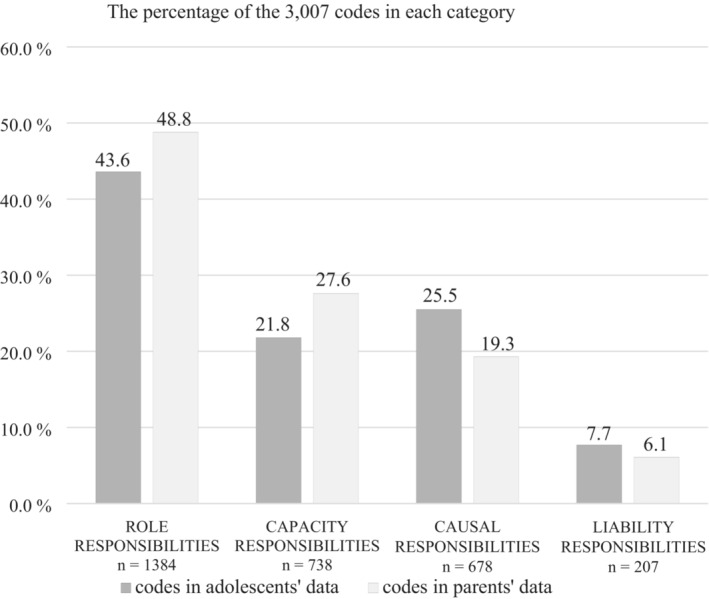
Responsibilities Expressed by Adolescents and Parents During the Interviews

Role responsibilities were the most frequent area of responsibility that were discussed by the parents and adolescent and liability responsibilities were the least discussed (Figure 2). The categories that were discussed were in the same order in both groups.

#### Role responsibilities

One fifth of the views that the adolescents expressed about role responsibilities focused on their own responsibilities and a little more than one fifth on their parents' responsibilities (Table 2). In contrast, the parents focused mainly on their own role responsibilities and much less on what their adolescents were responsible for. The adolescents spoke mainly about taking care of themselves independently in this category and this referred to their unquestionable duty to think about their health, safety, and future. Refusing offers of alcohol was one way that some adolescents looked after their own health. They were particularly likely to state that role responsibilities also meant loyally caring for their friends. Parents felt that adolescents needed to break away from them as they grew toward independence, learning to think for themselves and make personal decisions. This included whether they chose to use alcohol. But they also said that adolescents were still developing and still learning to take care of themselves.

**Table 2:** Views Related to the Four Categories of Responsibilities as a Result of the Deductive Content Analysis

Codes (%)	Adolescents' Descriptions	Sense of Responsibility		Parents' Descriptions	Codes (%)
**N = 1556**	Subcategory	Perspective	Perspective	Subcategory	**N = 1451**
**n = 688 (43.6%)**		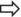 **ROLE RESPONSIBILITIES** 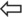			**n = 696 (48.8%)**
131 (19.0)	Taking care of themselves independently and autonomously	Adolescents' own role responsibilities, n = 321 (20.3%)	Adolescents' role responsibilities, n = 117 (8.2%)	Learning to take care of themselves independently	84 (12.1)
125 (18.2)	Taking care of, and supporting, their friends loyally, reciprocally and compassionately			Receiving information about alcohol use	22 (3.2)
65 (9.4)	Respecting and following the rules laid down by authorities			Loyally taking care of friends and stopping them getting into trouble	11 (1.6)
169 (24.6)	Guiding, educating and teaching adolescents to make rational and considered decisions	Parents' role responsibilities, n = 367 (23.3%)	Parents' own role responsibilities, n = 579 (40.6%)	Guiding and teaching adolescents by providing information	208 (29.9)
				Vigilantly monitoring adolescents around the clock	138 (19.8)
146 (21.2)	Providing the main authority figure that monitors and controls adolescents			Being a strong, determined and consistent authority figure	137 (19.7)
23 (3.3)	Looking after, and caring for, the adolescents			Serving as an appropriate role model with regard to alcohol use	67 (9.6)
				Looking after, helping and supporting adolescents with confidentiality, kindness and empathy	29 (4.2)
29 (4.2)	Serving as an appropriate role model with regard to alcohol use				
**n = 344 (21.8%)**		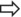 **Capacity responsibilities** 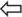			**n = 394 (27.6%)**
168 (48.8)	Making independent and positive decisions on alcohol use	Adolescents' own capacity responsibilities n = 280 (17.7%)	Adolescents' capacity responsibilities n = 206 (14.4%)	Developing independently and controlling own actions	149 (37.8)
112 (32.6)	Acting in accordance with decisions made with peers, friends and teachers			Acting in accordance with decisions made with peers and friends or with their support	57 (14.5)
48 (14.0)	Showing an interest in, and being aware of, what is going on in the adolescent's life	Parents' capacity responsibilities, n = 64 (4.1%)	Parents' own capacity responsibilities, n = 188 (13.2%)	Intervening in adolescents' alcohol use as appropriate	153 (38.8)
16 (4.7)	Trusting adolescents			Knowing when to seek timely help and support	35 (8.9)
**n = 403 (25.5%)**		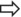 **Causal responsibilities** 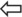			**n = 275 (19.3%)**
187 (50.0)	Not causing harmful consequences when drinking alcohol	Adolescents' own causal responsibilities, n = 256 (16.2%)	Adolescents' causal responsibilities, n = 60 (4.2%)	Not causing harmful consequences when drinking alcohol	31 (11.3)
69 (18.4)	Being told about harmful consequences honestly, openly and directly			Taking responsibility for consequences with peers	16 (5.8)
				Personally and openly taking responsibility for consequences	13 (4.7)
107 (28.6)	Reacting to, and managing, the consequences of drinking alcohol	Parents' causal responsibilities, n = 147 (9.3%)	Parents' own causal responsibilities, n = 215 (15.1%)	Acknowledging the harmful consequences of drinking alcohol on adolescents' health and future	90 (32.7)
33 (8.8)	Anticipating possible consequences			Reacting to the consequences of drinking alcohol and managing them	86 (31.3)
7 (1.9)	Identifying and noticing adolescents drinking alcohol				
				Identifying and noticing adolescents drinking alcohol	39 (14.2)
**n = 121 (7.7%)**		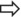 **Liability responsibilities** 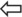			**n = 86 (6.1%)**
46 (38.0)	Taking legal responsibility for consequences related to drinking alcohol	Adolescents' own liability responsibilities, n = 80 (5.1%)	Adolescents' liability responsibilities, n = 21 (1.5%)	Taking legal responsibility for consequences related to drinking alcohol	15 (17.4)
34 (28.1)	Following the law			Following the law	6 (7.0)
32 (26.4)	Following the law	Parents' liability responsibilities, n = 41 (2.6%)	Parents' own liability responsibilities, n = 65 (4.6%)	Following the law	41 (47.7)
9 (7.4)	Taking legal responsibility for consequences			Taking legal responsibility for consequences	13 (15.1)
				Reporting adolescents' alcohol use to the authorities	11 (12.8)

Parents' role responsibilities referred to their tasks to raise, guide, and teach adolescents to make reasonable decisions and show discretion. Parents emphasized the importance of sharing the harm that alcohol can do openly, honestly, straightforwardly, and repeatedly. Both parties felt that parents are the main authority when it came to supervising and controlling adolescents. Parents said that they had a purposeful, consistent, and systematic authoritative position. It was their responsibility to know where their adolescents were 24 hours a day, so that they could protect them and safeguard their future and safety.

#### Capacity responsibilities

The adolescents focused on their own capacity responsibilities much more often than their parents' responsibilities. Parents focused on their adolescents' capacity responsibilities slightly more often than their own. Adolescents' capacity responsibilities were identified as developing their abilities to make independent and positive decisions. They described how they made shared decisions about alcohol with their peers, parents, or teachers and how they were responsible for abiding by any agreements they made. From parents' perspective, adolescents' capacity responsibilities comprised of their personal and developing abilities to control their actions, such as whether or not to use alcohol. Parents felt that adolescents and their peers had to be able to control their shared activities, such as whether they consumed alcohol, and this meant not getting drunk if they did drink alcohol.

Parents' capacity responsibilities referred to their interest in their adolescents' everyday lives and this was the driver for being aware of their whereabouts. Some adolescents said that parents had to trust their children and that some parents had given them permission to use alcohol based on trust. The parents' capacity responsibilities included their ability to intervene if the adolescent drank alcohol and this ability was stronger if they understood the harm that alcohol could do. Intervening involved talking and listening to their adolescents.

#### Causal responsibilities

Both groups highlighted their own causal responsibilities much more often than each other's. Adolescents viewed their causal responsibilities as not causing harmful consequences if the drank alcohol. This meant being completely abstinent or only using very small amounts of alcohol. If they failed to do this, they knew this would cause negative consequences, that risked their health and development and harmed their social relationships with parents and other people.

Parents' causal responsibilities referred to their duty to react and manage the harmful effects of their adolescents' alcohol use. Both parties felt that, in order to do this, parents needed to be able to anticipate and recognize alcohol use and its consequences. Parents also said they were responsible for stopping adolescents using alcohol and setting reasonable punishments. A few adolescents said it was more beneficial if parents supported and helped them, without judging or abandoning them.

#### Liability responsibilities

Adolescents and parents were more likely to identify their own liability responsibilities than those of the other party. Adolescents said that their liability responsibilities referred to legal responsibility for the consequences of their alcohol use, namely legal sanctions such as fines or child welfare notifications. They had to pay for any damage they had caused while they were using alcohol. They were also aware that they were not allowed to possess or use any alcohol at their age or provide it to others.

Parents recognized that their liability responsibilities were to follow the law. Some said this meant they could not supply or buy alcohol for their children, and they had to stop them from using it. However, some parents let their adolescents drink a little at weddings or mid‐summer parties. The aim of this was to make alcohol appear less tempting. Parents also recognized that their liability responsibilities included their legal responsibility to pay for any damage caused by their adolescents when they drank alcohol.

## DISCUSSION

### Need for Parental Involvement

This study produced descriptive and comparative knowledge on the responsibilities related to adolescents using alcohol, based on Hart's taxonomy. Half of the expressions used by the adolescents and parents related to the role responsibilities category and these focused on tasks that they were responsible for, in line with previous research.^2^, ^3^, ^24^ Adolescents felt they were responsible for looking after the health of themselves and their friends,^15^, ^26^ which suggests that they were aware of the harm that alcohol can cause. Sharing this knowledge is one of the main tasks that parents and school health care professionals need to undertake when they are promoting health literacy among adolescents.^8^, ^45^ Adolescents need to develop their health literacy, by acquiring and understanding information and applying the skills they have learnt.^8^ School health education could ensure that adolescents received more information about the impact of alcohol and this could encourage more responsible behavior and better choices about using alcohol.^46^, ^47^ School nurses could play a key role in this process.

The choices that adolescents made needed to be supported by their parents, whose role was to guide, educate, monitor and control their use of alcohol. This indicated that the role of parenting was valued.^30^, ^48^ Parents needed to be a responsible authority^49^ to stop adolescents to drink and this included providing rules that restricted alcohol use.^50^, ^51^

### Understanding Consequences and Similar Responsible Ideas

Adolescents understood that using alcohol had detrimental consequences. Acknowledging the consequences was also part of the parent's causal responsibility, even though previous research has described the ability to understand and anticipate the consequences of choices as a capacity responsibility.^2^, ^24^ Even without capacity responsibility, a person may be considered to have legal responsibility for the consequences of their actions.^24^ In this study, liability responsibilities included parents assuming the ultimate responsibility for the consequences and damage caused when their adolescents drank alcohol. The parents' willingness to take legal responsibility reflected their perceptions of their responsibilities.

Despite some slight differences, adolescents and parents tended to agree when it came to their views on responsibilities. Previous research has found similarities in adolescents' views on using alcohol and whether the way they acquired it was acceptable.^29^, ^52^ Adolescents had a more critical view than adults on alcohol poisoning.^29^ Preventive school health care could remind parents about what a continuous and challenging, but rewarding, responsibility it is to be involved and caring parents who support the growth of their adolescent child. This approach is also key to encouraging parents to increasingly guide adolescents toward taking responsibility for their own choices. Empowering and supporting adolescents to take their responsibilities has been shown to have a health‐promoting impact that prevents alcohol use.^5^

### Strengths and Limitations

One of the strengths of this study was that Hart's taxonomy provided a useful deductive approach for the secondary analysis of the data. The study emphasized the perspective of individuals and it is known that moral responsibilities develop in adolescent‐parent relationships.^53^ The taxonomy enabled us to identify and examine the various types of responsibility perceived by adolescents and parents. Using NVivo software helped us to achieve our research objectives, as this made the analysis process more systematic and helped us to increase the credibility. The uniformity of the secondary data, and their credibility with the qualitative original studies^28^, ^32^ confirmed their internal validity. The data collection and analysis process have been described in detail to enable transferability.

The limitation of deductive analysis is that it only takes the theory‐based structure of deductive domains into account.^54^ The credibility of our results may have been restricted by the fact that the data were not collected in a targeted manner based on the research questions used for this study. Some interpretation bias may have emerged from minor differences in the tone of the expressions,^35^ as there was no opportunity to ask for more detail, as this was a secondary analysis. For example, the term role responsibilities was not actually used in the interviews. The interviewees represented culturally and sociodemographically homogenous groups from a small area in a welfare state. As a result, the results cannot be widely generalized.

### Conclusions

Responsibilities are a key, multidimensional part of adolescents' alcohol use and this study found that they were related to the roles of adolescents and parents. We found that similar points were emphasized by both parties. Although adolescents are becoming increasingly aware of their own choices, and the consequences of those choices, parents continue to play an important role and bear the ultimate responsibility for their alcohol use. The key to preventing alcohol use in adolescents, and supporting healthy choices, is to discuss the adolescents' own role and responsibilities in detail and in concrete terms. Additional research is required to identify responsibilities around alcohol use from the perspective of school health services, including the role that school nurses could play. This will help to provide adolescents and parents with optimal support related to their responsibilities around alcohol use by adolescents.

## IMPLICATIONS FOR SCHOOL HEALTH

Questions about responsibilities play a key role when adolescents are making choices about alcohol. Understanding these responsibilities and incorporating them into school health care can improve the support that adolescents receive about healthy and positive choices.^15^, ^55^, ^56^ This study provides new knowledge about how adolescents and parents perceived their responsibilities when it came to adolescents using alcohol. We hope that our findings could help global school health care services when they are planning, implementing and monitoring health promotion campaigns that focus on adolescents' health and well‐being. School nurses could encourage adolescents to take responsibility for their own behavior by encouraging adolescents to think about how they would handle situations involving alcohol. These would include using their expertise as health professionals to help adolescents to develop the skills they need to react to complex situations. These are skills for creating and modifying roles for adolescents and balancing the right amounts of challenge and agency.^57^

Adolescents need support to discharge their responsibilities for healthy choices and school nurses can play a key role in discussing the adolescents' own roles and responsibilities with them. This could improve mutual understanding on this issue between the adolescents, their families, and their schools. School nurses could discuss issues individually with students during their annual health check‐ups. Group discussions could be another option, as peers play a central role in adolescents' lives. These would support the choices that adolescents make to enhance their health, including avoiding using alcohol. Positive communication between homes and schools is important. It is worth setting up mechanisms so that adolescents, parents and families can get consciously involved in discussions about responsibilities around alcohol. This mutual communication could include educational materials and information about adolescents' health. It would also be important to create greater awareness of how parents can become involved in school health activities and the importance of communicating with teachers and the school nurse.^58^

Responsibilities should form part of the health education curriculum in schools. For example, students could be encouraged to refuse alcohol by being made aware of the harmful health and legal consequences of drinking. Finnish laws on alcohol protect and empower adolescents^59^ and these can help them to make independent choices about whether they use alcohol or not.^60^

### Human Subjects Approval Statement

The Committee on Research Ethics of the University of Eastern Finland (Statement UEF/12/2017) approved this study.

### Conflict of Interest

The authors have no conflicts of interest to declare.

### Author Contributions

M.A.M., K.E.M., M.K.K: Study design. M.A.M: Data collection. M.A.M., K.E.M., M.K.K: Data analysis and writing manuscript. All the authors contributed to the analysis and revisions of the manuscript and all the authors read and approved the final version.
